# Mechanisims of asthma and allergic disease – 1085. Safety and tolerability of escalating doses of house dust mite peptide antigen desensitisation

**DOI:** 10.1186/1939-4551-6-S1-P81

**Published:** 2013-04-23

**Authors:** Roderick Peter Hafner, Mark Larché, Paul Laidler, Pascal Hickey, Jacques Hébert

**Affiliations:** 1 Circassia Limited, Oxford, UK; 2 Department of Medicine, Mcmaster University, Hamilton, ON, Canada; 3 Adiga Life Sciences Inc., Hamilton, ON, Canada; 4 Centre De Recherche Appliqué En Allergie De Québec, Québec City, QC, Canada

## Background

House Dust Mite (HDM) accounts for 20-25% of the allergic rhinoconjunctivitis disease burden worldwide. Previous studies have shown immunotherapy using peptides from Fel d 1 can induce tolerance in cat allergic subjects. This study identified T-cell epitopes derived from HDM allergens and evaluated their safety and efficacy in a clinical study.

## Methods

Potential T-cell epitopes were identified by algorithm, screened for ability to stimulate T-cell responses in ex-vivo blood samples from HDM allergic subjects and tested to confirm they did not cause basophil histamine release. A second group of HDM allergic subjects attended a challenge where Conjunctival Provocation Test (CPT) response and Early (EPSR) and Late Phase Skin Response (LPSR) were measured. Subjects were randomised to one of 5 cohorts of 10 subjects. In each cohort 8 subjects received HDM peptide antigen desensitisation (PAD) using the identified T-cell epitope mixture and 2 subjects received placebo. The first cohort received 4x0.03nmol 4weeks(wk) apart; successive cohorts received 4 administrations 4wk apart of 0.3, 1, 3 and 12nmol, respectively. EPSR, LPSR and CPT were re-measured 18-22wk after starting treatment.

## Results

HDM-PAD was safe and well tolerated with no Serious Adverse Events. The largest number of Treatment Emergent Adverse Events (TEAEs) occurred in the 0.03nmol group and the least in the 3nmol group. The most commonly reported TEAEs in subjects who received HDM-PAD were nasopharyngitis, influenza, gastroenteritis and nausea. There were no changes in mean FEV1 on dosing days for any dose of HDM-PAD or placebo. Subjects treated with four of the five HDM-PAD doses showed changes from baseline in CPT score at 18-22wk of between -16.7% to -41.4%, compared with no change for placebo. A statistically significant median %change from baseline in CPT score of -36.7% (p=0.0257 vsplacebo) and the largest change in EPSR (median %change -39.19%) and LPSR (median %change -51.19%) was observed after 3nmol HDM-PAD.

## Conclusions

HDM-PAD is safe and well tolerated when given as 4 intradermal injections 4wk apart, at doses up to 12nmol in HDM allergic subjects. Reductions in EPSR, LPSR and CPT after HDM-PAD indicate the identified T-cell epitopes have biological activity and merit further evaluation for treatment of HDM allergy.

